# The Impact of Functional Overreaching on Post-exercise Parasympathetic Reactivation in Runners

**DOI:** 10.3389/fphys.2020.614765

**Published:** 2021-01-08

**Authors:** Clint R. Bellenger, Rebecca L. Thomson, Kade Davison, Eileen Y. Robertson, Jonathan D. Buckley

**Affiliations:** ^1^Alliance for Research in Exercise, Nutrition and Activity (ARENA), Allied Health and Human Performance Unit, University of South Australia, Adelaide, SA, Australia; ^2^South Australian Sports Institute, Adelaide, SA, Australia

**Keywords:** heart rate, heart rate variability, athletic performance, autonomic nervous system, overload training

## Abstract

While post-exercise heart rate (HR) variability (HRV) has been shown to increase in response to training leading to improvements in performance, the effect of training leading to decrements in performance (i.e., overreaching) on this parameter has been largely ignored. This study evaluated the effect of heavy training leading to performance decrements on sub-maximal post-exercise HRV. Running performance [5 km treadmill time-trial (5TTT)], post-exercise HRV [root-mean-square difference of successive normal R-R intervals (RMSSD)] and measures of subjective training tolerance (Daily Analysis of Life Demands for Athletes “worse than normal” scores) were assessed in 11 male runners following 1 week of light training (LT), 2 weeks of heavy training (HT) and a 10 day taper (T). Post-exercise RMSSD was assessed following 5 min of running exercise at an individualised speed eliciting 85% of peak HR. Time to complete 5TTT likely increased following HT (*ES* = 0.14 ± 0.03; *p* < 0.001), and then almost certainly decreased following T (*ES* = −0.30 ± 0.07; *p* < 0.001). Subjective training tolerance worsened after HT (*ES* = −2.54 ± 0.62; *p* = 0.001) and improved after T (*ES* = 2.16 ± 0.64; *p* = 0.004). In comparison to LT, post-exercise RMSSD likely increased at HT (*ES* = 0.65 ± 0.55; *p* = 0.06), and likely decreased at T (*ES* = −0.69 ± 0.45; *p* = 0.02). A moderate within-subject correlation was found between 5TTT and post-exercise RMSSD (*r* = 0.47 ± 0.36; *p* = 0.03). Increased post-exercise RMSSD following HT demonstrated heightened post-exercise parasympathetic modulation in functionally overreached athletes. Heightened post-exercise RMSSD in this context appears paradoxical given this parameter also increases in response to improvements in performance. Thus, additional measures such as subjective training tolerance are required to interpret changes in post-exercise RMSSD.

## Introduction

The ability of coaches and sport science practitioners to readily and accurately predict athletic training status would assist in optimising training, since this information could be used to adjust training loads (Buchheit, 2014). Of particular importance in well-trained athletes, who often experience periods of high training stress, is the ability to detect the early onset of training-induced fatigue (i.e., functional overreaching) (Meeusen et al., 2013). Accurate identification of functional overreaching may ensure that training load is reduced to facilitate recovery and supercompensatory performance improvement, before the accumulation of training-induced fatigue gives rise to the more severe conditions of non-functional overreaching and overtraining, which can lead to extended periods (i.e., weeks to months) of attenuated performance (Meeusen et al., 2013).

Numerous psychological and physiological markers of training status have been evaluated over the years, however, their validity and/or practicality has yet to be fully established (Borresen and Lambert, 2009; Meeusen et al., 2013). Psychological measures such as the Profile of Mood Status questionnaire (McNair et al., 1971) and the Daily Analysis of Life Demands for Athletes questionnaire (Rushall, 1990) have been investigated extensively, however, the subjective nature of these assessments means that they are susceptible to manipulation for competition or training gain (Urhausen and Kindermann, 2002), are influenced by age and cognitive development (Groslambert and Mahon, 2006) and may be unreliable as a result of their reliance on memory recall (Shephard, 2003). Similarly, physiological parameters, including sub-maximal blood lactate concentrations, hormones and neuromotor control of movement have been shown to be altered by heavy overload training (Jacobs, 1986; Lehmann et al., 1992; Fuller et al., 2017; Greenham et al., 2018; Bellenger et al., 2019), however, their practicality and/or validity remains to be established as a result of either variable results, invasive assessment techniques or need for specialised/expensive equipment (Urhausen and Kindermann, 2002; Borresen and Lambert, 2009; Greenham et al., 2018).

The assessment of autonomic nervous system function has become a popular tool for predicting athletic training status (Buchheit, 2014). This is because the ANS interacts with many physiological systems (Aubert et al., 2003), and the ANS’s responsiveness to changes in training load may indicate the ability to adapt to an exercise stimulus (Borresen and Lambert, 2008). Specifically, research has focussed on predicting training status through autonomic heart rate (HR) regulation as it provides a simple, non-invasive measure of ANS function (Bosquet et al., 2008). Common measures of autonomic HR regulation include resting HR, submaximal HR, maximum HR, resting and post-exercise HR variability (HRV), HR Recovery (HRR) and HR acceleration (Borresen and Lambert, 2008; Buchheit, 2014; Bellenger et al., 2016a).

With regard specifically to post-exercise HRV, a review of the literature on autonomic HR regulation and athletic training showed that while a number of studies had investigated the effect of training leading to positive adaptations on post-exercise HRV (Bellenger et al., 2016a), only one study (Dupuy et al., 2013) had investigated the effect of overreaching training leading to negative adaptations on post-exercise HRV. This lack of research on the potential for post-exercise HRV to indicate negative training adaptation is surprising given that studies facilitating positive training adaptations showed increases in post-exercise parasympathetic modulation (Buchheit et al., 2008, 2010, 2011, 2012a,b), indicating the sensitivity of post-exercise HRV for detecting changes in training status in this context. Furthermore, the one study that has evaluated post-exercise HRV responses to overreaching training (Dupuy et al., 2013), did so after maximal exercise, which negates its practical application in athletes as it may be contraindicated to have an athlete exercise at maximal intensities if they are at risk of developing non-functional overreaching or overtraining, since this will only exacerbate the condition (Bellenger et al., 2016a). Additionally, given the aim of continuous monitoring of HR parameters is to predict training status (for which the gold standard assessment is maximal exercise performance), the assessment of any HR parameter during or following maximal performance is essentially redundant in practice since a measure of performance (i.e., time to complete a set distance, time to exhaustion, maximal aerobic power or speed) will also be measured (Bellenger et al., 2016a).

Consequently, the primary aim of this study was to evaluate the effect of heavy overload training leading to performance decrements, reflecting a state of functional overreaching, on sub-maximal post-exercise parasympathetic modulation. Additionally, Bellenger et al. (2016a) highlighted that previous studies assessing post-exercise HRV had done so during the final 3–5 min of a 5 min period of quiet rest (Buchheit et al., 2008, 2010, 2011, 2012a,b; Dupuy et al., 2013), and thus this study sought to examine whether the time-course of post-exercise parasympathetic modulation assessment could be reduced to aid practical application.

## Materials and Methods

### Participants

Fifteen male runners or triathletes were recruited from clubs in Adelaide, South Australia. Participants were eligible for inclusion if they displayed no known signs or symptoms of cardiometabolic disease, were currently completing at least 40 km of running per week, self-reported as injury free in the 3 months prior to undertaking the study, and could complete a 5 km treadmill time trial (5TTT) in less than 23 min. The University of South Australia’s Human Research Ethics Committee granted study approval and volunteers provided written informed consent prior to participating.

### Experimental Overview

In this study, individual specific running speeds corresponding to 65 and 85% of peak HR were used as the exercise stimulus prior to assessment of post-exercise HRV as a means of normalising the cardiovascular stress between individuals. This was ultimately performed to facilitate the assessment of a separate HR parameter (the maximal rate of heart rate increase, rHRI) at individual specific workloads, and results of this analysis have been published elsewhere (Bellenger et al., 2020). The current analysis therefore represents a secondary analysis of post-exercise HRV data from the study designed to evaluate rHRI that was unable to be reported in the aforementioned publication due to word limit restrictions. As such, the running performance and training-related variables assessed in the current study have also been published elsewhere (Bellenger et al., 2020). These same performance and training-related variables were required in the present study to demonstrate the effectiveness of the training intervention and to evaluate the sensitivity of post-exercise HRV for tracking training status (via correlation analysis).

Two pre-study familiarisation sessions allowed for quantification of the aforementioned individual specific running speeds. As described in greater detail in Bellenger et al. (2020), the linear relationship between running speed and HR at three submaximal workloads was first determined. Using the peak HR obtained during a 5TTT, running speeds corresponding to 65 and 85% of peak HR were identified. This process was repeated during both familiarisation sessions and the calculated running speeds were averaged. These speeds were then fixed and utilised at each testing visit thereafter, such that running speeds were constant within individuals, but different between individuals, in order to elicit a similar cardiovascular stress across all participants.

Following familiarisation, participants had their post-exercise HRV and 5TTT performance assessed after 1 week of light training (LT; baseline), 2 weeks of heavy training (HT; overreached state) and 10 days of Tapering (T; recovered and adapted state). Assessments occurred the day after completion of each period’s final training session. The impact of this training on daily resting HRV and markers of subjective training tolerance was also investigated.

### Post-exercise HRV Assessment and Calculation

To assess post-exercise HRV, participants ran for 10 min at the two speeds designed to elicit 65 and 85% of peak HR (5 min each) on a treadmill. At the cessation of this running task, participants sat upright in a chair for 5 min to allow for collection of post-exercise HR data.

RR interval data from this running task and associated recovery period were transferred to Polar Protrainer 5 software (Polar Electro Oy, Kempele, Finland) where artefacts or ectopic heart beats were removed using the manufacturer’s automatic filtering process. Data were then exported to HRV analysis software (Kubios HRV Analysis, version 2.0 beta 1, The Biomedical Signals Analysis Group, University of Kuopio, Finland) where any remaining artefacts or ectopic heart beats were manually removed. The root-mean-square difference of successive normal R-R intervals (RMSSD) has been advocated as the preferred index of HRV for the monitoring of athletic training status (Plews et al., 2013a; Buchheit, 2014; Bellenger et al., 2016a), and the natural logarithm of this index was consequently analysed as a measure of vagal-related HR modulation. Minutes 3–5 of the recovery period (i.e., 3 min of recording; Ln RMSSD_*mi*__*n*__3__–__5_) were analysed in accordance with the usual practice of evaluating post-exercise HRV between 3 and 5 min after exercise to allow for stationarity of the heart rate response (Buchheit et al., 2008, 2010, 2011, 2012a,b; Dupuy et al., 2013). In addition, minutes 3–4 (i.e. 2 min of recording; Ln RMSSD_*mi*__*n*__3__–__4_) and minute 3 (i.e., 1 min of recording; Ln RMSSD_*min*__3_) were also assessed to determine if post-exercise HRV assessment may be shortened to facilitate greater practical application. Post-exercise RR interval, Ln RMSSD and Ln RMSSD: RR interval were presented as values collected during testing visits at the end of LT, HT and T.

### Running Performance Assessment

Post-exercise HR testing was followed by a 5TTT where the time taken to run 5 km on a motorised treadmill was recorded as the measure of exercise performance. As previously reported (Bellenger et al., 2020), participants chose their preferred starting speed during familiarisation which remained constant across visits. Participants were blinded to running time and speed, but were free to adjust the treadmill speed as desired to complete 5 km in the fastest time possible. Reliability of the 5TTT in a separate group of well-trained runners was determined to be excellent (*CV* = 1.3%, Fuller et al., 2015).

### Resting Heart Rate Variability Assessment and Calculation

RR intervals during 3 min of quiet rest were recorded every morning at home upon wakening and after emptying the urinary bladder for assessment of resting HRV. A standing posture was adopted for this assessment based on literature demonstrating enhanced sensitivity of this posture over supine measures (Le Meur et al., 2013; Bellenger et al., 2016a,b).

Minutes 2 and 3 of the RR interval data from these morning-waking assessments were analysed in the same means as described previously for post-exercise HRV. Morning-waking RR interval, Ln RMSSD and Ln RMSSD: RR interval were analysed as a rolling 7 day average and presented as values on the final days of LT, HT, and T.

### Training Tolerance Assessment

As previously reported (Bellenger et al., 2020), subjective measures of training tolerance were determined throughout the training intervention via a Daily Analysis of Life Demands for Athletes (DALDA) questionnaire, which has been shown to detect perturbations in various parameters (e.g., diet, social/work life, sleep, fatigue, muscle soreness, etc.) resulting from periods of overload training in athletes (Halson et al., 2002; Bellenger et al., 2016b). The DALDA was scored on a three-point scale (worse than normal, normal, better than normal).

### Training Intervention

Peak HR determined during familiarisation was used to prescribe training intensities. LT required 7 days of running exercise for 30 min per day at 65–75% of peak HR, such that it would allow participants to be rested and recovered from any pre-study training before starting HT. During HT, participants were required to complete 14 days of running exercise for 66 min per day, with 36% of the training performed above 88% of peak HR, and was intended to induce substantial fatigue from which participants would not recover by the day after the final training session. Specific details of the HT interval program have been provided previously (Bellenger et al., 2016b). Following HT, participants completed 10 days of tapering, with rest on days 1 and 9. Seven of the eight training sessions during T required 30 min per day at 65–75% of peak HR, with one interval session (four repeats of 3 min at 69–81% peak HR followed by 2 min at 88–92% peak HR) conducted on day seven to provide participants with variety in training during this training phase. HR data were recorded at 15 s intervals during training for determination of training load using Training Impulse (TRIMP) (Banister, 1991) (duration in minutes multiplied by% of peak HR).

### Statistical Analysis

Data were analysed using PASW Statistics 18.0 (SPSS, Chicago, IL, United States) and presented as mean ± SD, and effect sizes (ES) ± 90% confidence intervals. Data were log transformed before analysis to reduce bias from non-uniformity of error (Hopkins et al., 2009). Outcome measures were compared using repeated measures analysis of variance with Bonferroni *post hoc* comparison and statistical significance set at *p* < 0.05. Data were also analysed using magnitude-based inferences (Hopkins et al., 2009), with changes in variables after each training period analysed using a modified statistical spreadsheet (Hopkins, 2006), which calculated ES between time-points of interest using pooled standard deviation (Cohen, 2010). Threshold values for ES statistics were ≤ 0.2 (trivial), >0.2 (small), >0.6 (moderate), >1.2 (large), >2.0 (very large), and >4.0 (extremely large) (Hopkins et al., 2009). Probabilities to establish whether the true (unknown) differences were lower, similar, or higher than the smallest worthwhile change were also calculated. Chances of higher or lower differences were evaluated qualitatively as: <1%, almost certainly not; 1–5%, very unlikely; 5–25%, unlikely; 25–75%, possibly; 75–95%, likely; 95–99%, very likely; and >99%, almost certain. If the chance of higher and lower differences was >5%, the true difference was assessed as unclear. Within-subject correlations between HR and performance variables across testing time-points were evaluated using univariate analysis of covariance (Bland and Altman, 1995), with r values evaluated as: 0.0–0.1, trivial; 0.1–0.3, small; 0.3–0.5, moderate; 0.5–0.7, large; 0.7–0.9, very large; 0.9–1.0, nearly perfect (Hopkins et al., 2009). Absolute agreement between Ln RMSSD_*min*__3_, Ln RMSSD_*min*__3__–__4_ and Ln RMSSD_*min*__3__–__5_ was determined through limits of agreement analysis (Bland and Altman, 2010), while relative agreement was determined using the intra-class correlation (ICC).

## Results

As previously reported (Bellenger et al., 2020), 14 of the 15 recruited participants completed the study (age 35.8 ± 10.0 years; height 1.78 ± 0.09 m; body mass 77.3 ± 10.0 kg). The participant who did not complete was unable to tolerate the demands of HT and withdrew during this phase of the study. Three of the 14 completed participants were diagnosed as acutely fatigued, but not overreached, as they did not experience a decline in 5TTT performance that was greater than the natural variability in this test [i.e., CV = 1.3% (Fuller et al., 2015)] (Le Meur et al., 2014), and were excluded from further analysis so as not to attenuate analysis of the true effect of HT on variables of interest in those participants experiencing functional overreaching as recommended by Bellenger et al. (2016a). A sub-group analysis was not performed on these three participants given the small sample size. Thus, data for 11 participants were included for analysis (age 37.5 ± 8.2 years; body mass 78.5 ± 10.3 kg; self-reported weekly running distance 46.2 ± 16.8 km in the previous 6 months).

### Training Impulse, 5 km Treadmill Time-Trial and Peak Heart Rate

Table 1 shows the average daily TRIMP, 5TTT performance and peak HR throughout the training intervention. As previously reported (Bellenger et al., 2020), daily TRIMP almost certainly increased (ES ± 90% confidence interval = 3.85 ± 0.77; *p* < 0.001) at HT, and was accompanied by a very likely increase in time taken to complete 5TTT (0.14 ± 0.03; *p* < 0.001) and an almost certain decrease in peak HR (−0.75 ± 0.24; *p* = 0.001). In comparison to HT, daily TRIMP almost certainly decreased at T (−5.78 ± 0.71; *p* < 0.001), while 5TTT almost certainly decreased (−0.30 ± 0.07; *p* < 0.001) and peak HR almost certainly increased (0.72 ± 0.22; *p* < 0.001).

**TABLE 1:** Effect of training intervention on outcomes of interest.

	**LT**	**HT**	**T**
**Daily TRIMP (AU)**	2740 (301)	5182(890)*	2028(407)*^#^
**5TTT (min:s)**	19:35(2:21)	19:57(2:21)*	19:09(2:13)*^#^
**Peak HR (bpm)**	184 (11)	177(9)*	184(8)^#^
**DALDA (AU)**	1.3 (1.0)	7.3(3.4)*	2.2(2.1)^#^

Values are mean (standard deviation). AU, arbitrary units; bpm, beats per minute; DALDA; number of “worse than normal” scores on the Daily Analysis of Life Demands for Athletes questionnaire; HR, heart rate; HT, heavy training; LT, light training; min:s, minutes:seconds; TRIMP, training impulse; T, taper training; 5TTT, 5 km treadmill time-trial. *, significantly different to LT (p < 0.05); #, significantly different to HT (p < 0.05).

### Training Tolerance

As previously reported (Bellenger et al., 2020), the number of “worse than normal” scores on the DALDA was 1.3 ± 1.0 at LT. DALDA “worse than normal” scores were almost certainly increased at HT (2.54 ± 0.62; *p* = 0.001), and then almost certainly decreased at T (−2.16 ± 0.64; *p* = 0.004; Table 1).

### Resting Heart Rate Variability

**FIGURE 1:**
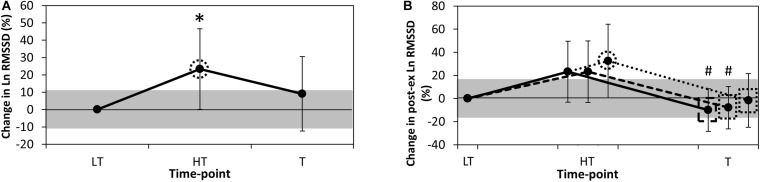
Percentage change in **(A).** Resting Ln RMSSD and **(B).** Post-exercise Ln RMSSD from LT. Data are mean ± 90% confidence level. HT, heavy training; Ln RMSSD, natural logarithm of the root-mean-square difference of successive normal R-R intervals; LT, light training; T, taper training. Grey shaded areas represent the smallest worthwhile change for each variable. Continuous line **(B)**, analysis of post-exercise recording minutes 3–5; dashed line **(B)**, analysis of post-exercise recording minutes 3–4; dotted line **(B)**, analysis of post-exercise recording minute 3; dotted circle, likely chance of practically meaningful difference in value from LT; dotted rectangle, likely chance of practically meaningful difference in value from HT; dashed rectangle, very likely chance of practically meaningful difference in value from HT; *, significantly different to LT (*p* < 0.05); #, significantly different to HT (*p* < 0.05).

Standing values of RR interval, Ln RMSSD and Ln RMSSD: RR interval were 859 ± 94 ms, 3.40 ± 0.29 ms and 3.99 ± 0.47 units, respectively, at LT. Ln RMSSD likely increased at HT in comparison to LT (0.53 ± 0.47; *p* = 0.04), while changes at other time-points were assessed as possible or very likely trivial (≤0.31 ± 0.31; *p* ≥ 0.21; Figure 1A). Changes in RR interval and Ln RMSSD: RR interval throughout the intervention were statistically significant (≤0.57 ± 0.22; *p* ≤ 0.006), but were assessed as likely trivial to almost certainly trivial when contextualised by their respective SWCs.

### Steady-State Heart Rate During Exercise

The mean running speed during assessment of post-exercise HR parameters was 9.2 ± 1.5 km/h (range 8.0–12.0 km/h) for the first stage, and 14.0 ± 2.2 km/h (range 11.5–18.5 km/h) for the second. At LT, steady-state HR was 84.51 ± 1.88% of peak HR, which almost certainly decreased at HT in comparison to LT (−1.43 ± 0.53; *p* = 0.002), and very likely remained decreased at T in comparison to LT (−0.95 ± 0.43; *p* = 0.007).

### Post-exercise Heart Rate Variability

Post-exercise Ln RMSSD_*min*__3_, Ln RMSSD_*min*__3__–__4_ and Ln RMSSD_*m*__*in*__3__–__5_ were 3.21 ± 0.36, 3.24 ± 0.35, and 3.22 ± 0.33 ms, respectively, at LT. Post-exercise Ln RMSSD_*min*__3_ likely increased at HT in comparison to LT (0.65 ± 0.55; *p* = 0.06), while Ln RMSSD_*mi*__*n*__3__–__4_ and Ln RMSSD_*min*__3__–__5_ were possibly increased at this time-point (0.48 ± 0.47; *p* = 0.11 and 0.47 ± 0.46; *p* = 0.10, respectively). At T, all durations of post-exercise Ln RMSSD likely to very likely decreased in comparison to HT (−0.69 ± 0.45; *p* = 0.02, −0.64 ± 0.32; *p* = 0.01, and −0.71 ± 0.33; *p* = 0.003, respectively, Figure 1B). Differences in the response to training between post-exercise Ln RMSSD_*min*__3_, Ln RMSSD_*mi*__*n*__3__–__4_ and Ln RMSSD_*min*__3__–__5_ were unclear (≤0.22 ± 0.72; *p* ≥ 0.24).

Changes in all durations of post-exercise RR interval and Ln RMSSD: RR interval throughout the intervention were statistically significant (≤0.85 ± 0.33; *p* ≤ 0.006), but were assessed as likely trivial to almost certainly trivial when contextualised by their respective SWCs.

### Agreement Between Measures of Post-exercise Heart Rate Variability

Differences between post-exercise Ln RMSSD_*min*__3__–__5_, Ln RMSSD_*min*__3__–__4_ and Ln RMSSD_*min*__3_ were likely trivial to almost certainly trivial (≤0.18 ± 0.22; *p* ≥ 0.22; Table 2) across the testing timepoints. Limits of agreement analysis indicated that the precision of the difference between Ln RMSSD_*min*__3_ and each of Ln RMSSD_*min*__3__–__5_ and Ln RMSSD_*min*__3__–__4_ was greater than the coefficient of variation for this parameter (i.e., 15.7%, Al Haddad et al., 2011), such that a practically meaningful difference between these recording durations may be evident. ICCs were very large to almost perfect across LT, HT and T (*r* ≥ 0.87).

**TABLE 2:** Agreement between post-exercise heart rate variability assessments.

**Comparison**	**Variable**	**LT**	**HT**	**T**
**Post-exercise Ln RMSSD_*min*__3__–__5_ vs. Ln RMSSD_*min*__3__–__4_ (ms)**	ICC	0.99	0.99	0.99
	Absolute bias (min3–4 − min3–5)	0.02	0.02	0.04
	% bias (min3–4 − min3–5)	2.07	2.02	4.46
	Absolute LOA	±0.13	±0.19	±0.11
	% LOA	±13.08	±19.87	±11.65
**Post-exercise Ln RMSSD_*min*__3__–__4_ vs. Ln RMSSD_*min*__3_ (ms)**	ICC	0.91	0.95	0.94
	Absolute bias (min3 − min3–4)	–0.03	0.05	0.04
	% bias (min3 − min3–4)	–2.73	4.62	3.91
	Absolute LOA	±0.33	±0.33	±0.32
	% LOA	±35.66	±35.67	±35.27
**Post-exercise Ln RMSSD_*min*__3__–__5_ vs. Ln RMSSD_*min*__3_ (ms)**	ICC	0.87	0.91	0.91
	Absolute bias (min3 − min3–5)	–0.01	0.07	0.08
	% bias (min3 − min3–5)%	–0.71	6.74	8.55
	Absolute LOA	±0.38	±0.42	±0.37
	% LOA	±42.41	±46.71	±41.05

*HT, end of heavy training testing visit; ICC, intra class correlation; Ln RMSSD, natural logarithm of the root-mean-square difference of successive normal R-R intervals; LOA, limits of agreement; LT, end of light training testing visit; _*min*__3__–__5_, heart rate variability measurement performed on data collected during the third and fifth minutes of a 5 min recovery period; _*min*__3__–__4_, heart rate variability measurement performed on data collected during the third and fourth minutes of a 5 min recovery period; _*min3*_, heart rate variability measurement performed on data collected during the third minute of a 5 min recovery period; ms, milliseconds; T, end of tapering testing visit.*

### Correlations

Within-subject analysis (using LT, HT, and T) revealed a large inverse correlation between 5TTT performance and 5TTT peak HR (*r* = −0.67; *p* < 0.001). Moderate positive correlations were found between 5TTT and post-exercise Ln RMSSD at minute 3 (*r* = 0.40; *p* = 0.06), minutes 3–4 (*r* = 0.47; *p* = 0.03) and minutes 3–5 (*r* = 0.49; *p* = 0.02). Moderate to large positive correlations were also found between resting Ln RMSSD and post-exercise Ln RMSSD at minute 3 (*r* = 0.45; *p* = 0.03), minutes 3–4 (*r* = 0.47; *p* = 0.02) and minutes 3–5 (*r* = 0.55; *p* = 0.01).

## Discussion

The primary finding in this study was heightened post-exercise parasympathetic modulation in functionally overreached athletes. Additionally, this heightened parasympathetic modulation was able to be detected over a period of time shorter than that previously assessed, which may aid the practical application of post-exercise HRV assessment for monitoring athletes.

The performance impairment induced by heavy overload training in the present study was subsequently followed by supercompensatory performance improvements after a period of taper. These changes in running performance were first accompanied by small to moderate increases in post-exercise parasympathetic modulation as assessed by Ln RMSSD, followed by moderate reductions in this parameter. Resultantly, the present study observed moderate positive within-subject correlations between running performance and post-exercise parasympathetic modulation, indicating that the heightened post-exercise parasympathetic modulation seen in the fatigued state explained at least some of the attenuated running performance at this timepoint. The finding of increased post-exercise parasympathetic modulation in a fatigued state may be considered paradoxical given that this parameter has also been shown to increase in athletes experiencing improvements in performance (Bellenger et al., 2016a). In the context of improved performance, increased post-exercise parasympathetic modulation was considered a positive adaptation to training since it indirectly indicated an enhanced ability to return to homeostasis following exercise. However, in the context of fatigue leading to attenuated performance, increased post-exercise parasympathetic modulation may be a consequence of the heightened *resting* parasympathetic modulation also shown in the present study and in a number of other recent studies (Le Meur et al., 2013; Bellenger et al., 2016b, 2017), ultimately indicating an overall parasympathetic “hyperactivity” in the autonomic regulation of HR by the ANS. While the exact mechanism by which the increased resting parasympathetic modulation occurs under fatigue is not fully known at present, it is hypothesised to limit the engagement of the sympathetic nervous system during exercise, which is supported by the reduction in peak HR observed in the present study and other overreaching studies (Achten and Jeukendrup, 2003; Bosquet et al., 2008; Le Meur et al., 2013, 2014; Bellenger et al., 2016a,b, 2017), likely attenuating cardiac output at maximal intensities and thereby decreasing exercise performance capacity (Le Meur et al., 2013).

Given that the post-HT testing visit occurred within 24 h of the final HT session, it may be speculated that the increase in post-exercise Ln RMSSD is an acute response to exercise, rather than a chronic response to HT. Greater resolution in the post-exercise Ln RMSSD data would be required to confirm or refute this speculation, however, the results of Bellenger et al. (2016b) suggest that increases in *resting* Ln RMSSD are the result of a cumulative, chronic impact of heavy overload training. Specifically, Bellenger et al. (2016b) used a rolling 7 day average of morning-waking Ln RMSSD assessments to show trivial (as denoted by ES) changes in resting Ln RMSSD on days 1–7 of HT, small changes on days 8–10 and finally moderate changes on days 11–14. Importantly, a similar cumulative pattern of change occurred in resting Ln RMSSD in the present study (data not reported), indicating that HT induced a chronic increase in resting Ln RMSSD. Since resting Ln RMSSD was correlated with post-exercise Ln RMSSD in the present study, it may be concluded that HT also induced a chronic increase in post-exercise Ln RMSSD.

Together, the paradoxical increases in resting and post-exercise parasympathetic modulation under conditions of fatigue further highlight the need for additional measures, such as the quantification of training load and an athlete’s subjective tolerance of that training load, to contextualise these increases and aid appropriate interpretation. This study adds further support to the utilisation of the DALDA questionnaire in this context (Bellenger et al., 2016b, 2017).

The present study also informs on the methodology of post-exercise parasympathetic modulation assessment. Specifically, the assessment of this parameter during the third minute of post-exercise recovery elicited a similar response to assessments at minutes 3–4 and 3–5. While precision of bias (i.e., limit of agreement analysis) between the three analysis durations indicated that Ln RMSSD_*min*__3_ may give a practically different value to each of Ln RMSSD_*min*__3__–__5_ and Ln RMSSD_*min*__3__–__4_, the very large to nearly perfect ICCs indicated that the between-participant ordering of these values was consistent for each recording duration, such that the response to training was similar. Consequently, assessment of parasympathetic modulation in the third minute following exercise may be utilised to detect changes in training status, which may subtly reduce the time burden associated with using post-exercise HR kinetics for monitoring athletic training.

As a result of the training intervention being performed by the participants outside of the laboratory, post-exercise HRV assessments were conducted only at the scheduled laboratory visits following each phase of training. Given the natural variation in day-to-day measures of post-exercise RMSSD (%*CV* = 15.7%, Al Haddad et al., 2011), training-induced changes in this parameter may be attenuated by a diminished signal-to-noise ratio. Consequently, it may be hypothesised that taking more than one measure per week, and calculating a weekly average of these measures could improve the signal-to-noise ratio, thereby increasing the sensitivity of post-exercise RMSSD assessment for detecting meaningful changes in training status. Utilising weekly averages in this context has previously been demonstrated in measures of resting HRV (Le Meur et al., 2013; Plews et al., 2013b; Bellenger et al., 2016b), where Plews et al. (2014) also showed that at least three measures per week were required. Thus, three or more assessments of post-exercise RMSSD in response to a standardised workload, perhaps performed as a warm-up prior to main training sets, may improve the sensitivity, and therefore the applicability, of post-exercise HRV for the practical monitoring of athletes in the field.

## Conclusion

The results of this study demonstrate heightened post-exercise parasympathetic modulation in functionally overreached athletes. However, this heightening appears paradoxical given the results of earlier research, and therefore emphasises the need for additional measures, such as subjective training tolerance, to allow for effective monitoring of athletic training. The present study also demonstrated that post-exercise parasympathetic modulation may be determined over a period of time shorter than that previously assessed, potentially enhancing the practical application of this HR parameter for athletic monitoring.

## Data Availability Statement

The raw data supporting the conclusions of this article will be made available by the authors, without undue reservation, to any qualified researcher.

## Ethics Statement

This study involving human participants was reviewed and approved by the University of South Australia Human Research Ethics Committee. The patients/participants provided their written informed consent to participate in this study.

## Author Contributions

CB conceived, designed the research project, conducted experiments, analysed data, and wrote the manuscript. CB, RT, KD, ER, and JB interpreted the data, drafted, and approved the manuscript. All authors contributed to the article and approved the submitted version.

## Conflict of Interest

The authors declare that the research was conducted in the absence of any commercial or financial relationships that could be construed as a potential conflict of interest.
